# Differential reproductive investment in co-occurring oviparous and viviparous common lizards (*Zootoca vivipara*) and implications for life-history trade-offs with viviparity

**DOI:** 10.1007/s00442-019-04398-w

**Published:** 2019-05-06

**Authors:** Hans Recknagel, Kathryn R. Elmer

**Affiliations:** Institute of Biodiversity, Animal Health & Comparative Medicine, College of Medical, Veterinary & Life Sciences, University of Glasgow, Glasgow, G12 8QQ UK

**Keywords:** Reproductive investment, Live-bearing, Egg-laying, Oviparity, Viviparity, Reptile, Squamate, Body size, Fitness, Alternative life-history trade-offs

## Abstract

Live-bearing reproduction (viviparity) has evolved from egg-laying (oviparity) independently many times and most abundantly in squamate reptiles. Studying life-history trade-offs between the two reproductive modes is an inherently difficult task, as most transitions to viviparity are evolutionarily old and/or are confounded by environmental effects. The common lizard (*Zootoca vivipara*) is one of very few known reproductively bimodal species, in which some populations are oviparous and others viviparous. Oviparous and viviparous populations can occur in sympatry in the same environment, making this a unique system for investigating alternative life-history trade-offs between oviparous and viviparous reproduction. We find that viviparous females exhibit larger body size, smaller clutch sizes, a larger reproductive investment, and a higher hatching success rate than oviparous females. We find that offspring size and weight from viviparous females was lower compared to offspring from oviparous females, which may reflect space constraints during pregnancy. We suggest that viviparity in common lizards is associated with increased reproductive burden for viviparous females and speculate that this promoted the evolution of larger body size to create more physical space for developing embryos. In the context of life-history trade-offs in the evolution of viviparity, we suggest that the extent of correlation between reproductive traits, or differences between reproductive modes, may also depend on the time since the transition occurred.

**Electronic supplementary material:**

The online version of this article (10.1007/s00442-019-04398-w) contains supplementary material, which is available to authorized users.

## Introduction

Live-bearing reproduction is one of the most ubiquitous life-history transitions across the animal kingdom (Sites et al. 2011). It has evolved independently from egg-laying more than 150 times across all vertebrates (Shine 2005; Blackburn 2006) and numerous times among invertebrates (Clutton-Brock 1991; Blackburn 1999). While the causes for the evolution of viviparity are not fully understood, recent advances in phylogenetic reconstruction and environmental data collection have shed light on this question in some taxonomic groups. Several studies now suggest that squamate reptiles, the group with the largest number of transitions from oviparity to viviparity, mainly evolved viviparity in response to cool climates (Tinkle and Gibbons 1977; Lynch 2009; Schulte and Moreno-Roark 2010; Lambert and Wiens 2013; Watson et al. 2014). Experimental case studies of squamates also support this hypothesis (Rodríguez-Díaz and Braña 2012), though many examples exist of tropical viviparous species (Tinkle and Gibbons 1977; Vitt and Blackburn 1983; Webb et al. 2006), which suggests other life-history trade-offs are important (Webb et al. 2006). In other animal groups, the causes are even less understood, mainly due to the limited number of transitions and the difficulty to separate correlative variables from causative factors (Wourms and Lombardi 1992; but see Bassar et al. 2014).

The evolution of viviparity entails dramatic changes in morphology, physiology, ecology and behaviour (Guillette 1993; Thompson and Speake 2006). Viviparity offers several potential fitness advantages, including protection of the embryo from adverse environmental conditions and predation, and higher trophic level at independence due to larger offspring size. The disadvantages of live-bearing include lower reproductive output of the female due to space constraint and reduced number of clutches, and increased female mortality due to limited locomotion and increased predation pressure (Wourms and Lombardi 1992; Blackburn 1999; Shine 2002; Sites et al. 2011). For example, it has been suggested that viviparous females tend to exhibit reduced clutch sizes, due to space constraint and a decrease in female locomotion ability with the number of offspring carried, relative to oviparous females (Seigel and Fitch 1984; Qualls and Shine 1995). Presumably to counteract the space constraint and the increase in predation pressure, some viviparous species evolved larger body sizes (Qualls and Shine 1995; Goodwin et al. 2002). Another adaptation that constitutes a trade-off with the decrease in clutch size is increased offspring survival. This can be achieved by larger offspring size at birth compared to progeny hatching from eggs, enhancing the survival of the offspring by increasing independence and avoiding predation (Goodwin et al. 2002). Viviparous species can also exhibit longer life spans. This allows them to produce more offspring across years and accounts for the lower reproductive output per season compared to oviparous species (Tinkle et al. 1970). Some support for these hypotheses comes from a few large-scale studies (Seigel and Fitch 1984; Stearns 1984; Meiri et al. 2012), although confounding phylogenetic and environmental effects have a substantial impact on life-history evolution associated with reproductive mode (Dunham and Miles 1985; Meiri et al. 2012; Bassar et al. 2014).

**Fig. 1:**
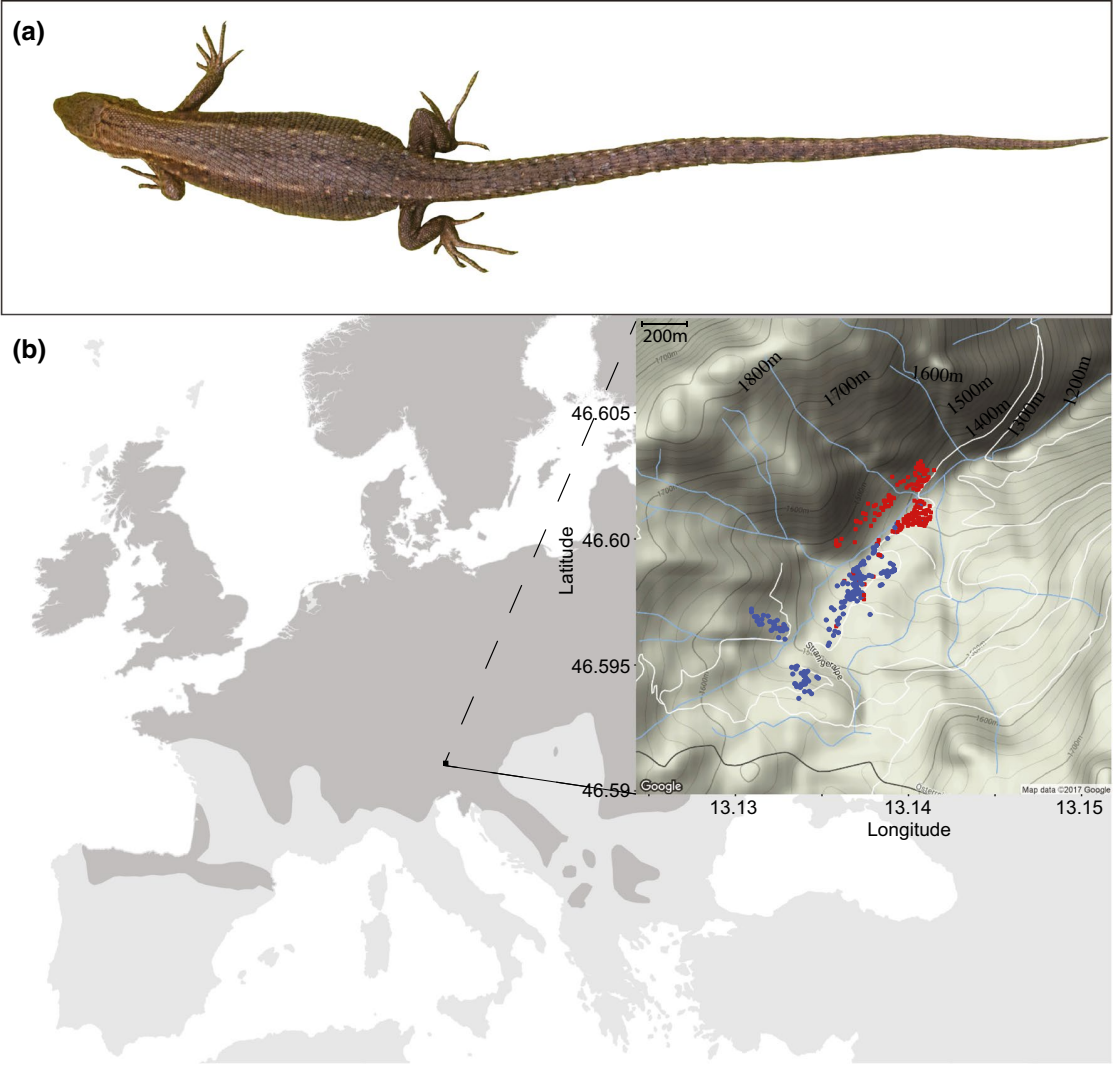
The **a** study organism: a female common lizard (*Zootoca vivipara*). The **b** distribution of the common lizard across Europe (dark grey shaded area, extracted from IUCN database). The sampling location situated in the Carinthian Alps in Austria is indicated in detail. The collection location for each female is indicated with red squares (oviparous) or blue dots (viviparous) (*N* = 438)

Common lizards (*Zootoca vivipara*) (Fig. 1a) are one of the few known animal species that are both oviparous or viviparous, with different intraspecific lineages being fixed for either reproductive mode (Fig. 1a; Guillette 1993; Mayer et al. 2000; Surget-Groba et al. 2006; Recknagel et al. 2018). These distinct phylogeographic lineages diverged between 2 and 4 mya (Surget-Groba et al. 2006). The two reproductive modes are usually allopatric and interbreed exceedingly rarely, even when they do come into contact (Lindtke et al. 2010; Cornetti et al. 2015a, b). Experimental studies using lizard enclosures have amassed substantial knowledge about reproductive traits and strategies within both oviparous (Gonzalez-Jimena and Fitze 2012; Breedveld and Fitze 2015; San-Jose et al. 2016) and viviparous populations (Boudjemadi et al. 1999; Fitze and Le Galliard 2008; Cote et al. 2008; Bleu et al. 2011; Richard et al. 2012; Bestion et al. 2015; Josserand et al. 2017; Dupoué et al. 2017a, b) in different geographic settings. Experimental crosses between oviparous and viviparous individuals have shown that reproductive mode is a purely genetically heritable trait (Arrayago et al. 1996).

Since oviparous and viviparous common lizards are usually found in different geographic regions, little is known about the functional ecology of alternative reproductive strategies associated with reproductive modes in a controlled, similar environment. We have been studying one of the few locations where the two reproductive lineages are found syntopically (Lindtke et al. 2010). This allows us to directly study reproductive effort in both modes in situ while minimizing confounding effects of environment, phylogeny, and plasticity (Sorci et al. 1996; Sorci and Clobert 1999; Lorenzon et al. 2001; Roitberg et al. 2013). These factors make common lizards an ideal model organism to test ecological and evolutionary hypotheses on alternative life-history trade-offs between the two reproductive modes (Blackburn 2006; Murphy and Thompson 2011).

Here, we tested five predictions on the trade-offs associated with reproductive mode in female common lizards. More specifically, we here refer to trade-offs as alternative life-history strategies between reproductive modes, in which life-history traits are presumed to be associated with fitness and reproductive success. These alternative trade-offs follow from theoretical expectations in life-history tactics. Specifically, that viviparous individuals exhibit (i) larger body size, (ii) decreased clutch size, (iii) larger offspring at birth, (iv) larger reproductive investment and (v) higher hatching success. Support for all predictions would indicate that viviparous common lizards have optimized their reproductive traits following life-history theory predictions (Tinkle 1969; Stearns 1976, 1992; Roff 1992). Alternatively, partial support for some of the predictions would indicate that factors such as cavity size (Qualls and Shine 1995) or offspring survival (Reznick 1982; Pike et al. 2008) could be limiting the viviparous common lizard’s reproductive output.

## Materials and methods

### Study site and species

The study was carried out in the Carinthian Alps in the Gailtal valley of Austria (Fig. 1b). Co-occurring viviparous and oviparous common lizards are extremely rare and to date this is the only known locality where both forms co-occur in high densities (Surget-Groba et al. 2006; Lindtke et al. 2010; Cornetti et al. 2015a, b). The study site covers an area of approximately 0.3 km^2^ and an altitude range of 200 m from 1380 to 1580 masl. Female common lizards were collected between April and August from 2013 to 2016 and caught by hand. Females were distinguished from males by the absence of a hemipenal bulge at the base of the tail. A female’s reproductive mode was assessed based on the number of days the clutch incubated after parturition/oviposition and hatching (viviparous = 0 incubation days, oviparous > 28 incubation days), the characteristics of the eggshell it laid, and genetic ancestry (Recknagel et al. unpubl.). Hybrid females (number of incubation days = 4–28) were excluded from the analysis (Lindtke et al. 2010). For each female, the location of capture and altitude was recorded. On average, oviparous individuals are found around an altitude of 1413 masl and viviparous around 1475 masl. The presence of a biting mark on the female’s belly or flank resulting from mating served to identify whether she was pregnant. All lizards were weighed using a smart weigh high precision scale (to the nearest 0.001 g) and measured for snout-vent length (SVL) and tail length (TL) using digital callipers (to the nearest 0.01 mm) immediately after capture. In 2016, female lizards were weighed a second time after oviposition/parturition.

### Reproductive traits

Pregnant females (*N* = 438) were kept until oviposition or parturition to assess their reproductive mode and other reproductive traits. On average, oviparous females were kept for 24.5 days in captivity, and viviparous for 25.2 days. Females were individually housed in 56 × 39 × 28 cm plastic terraria with netting on top and one side to guarantee air flow. All terraria were set up within tents close to the study area (at ~ 900 masl), so that lizards were exposed to natural temperature variation. Tents (‘Event Tent’ by Vango) included plastic windows that allowed for insolation and screening for air flow. Each terrarium contained sand as substrate, shelters (pieces of wood), moisturized moss, and a bowl of water. Insolation and shelters providing shade allowed lizards to thermoregulate, providing a temperature range close to what they would experience in their natural environment at the sampling site. Lizards were fed ad libitum with mealworms (*Tenebrio molitor*) and crickets (*Gryllus assimilis*). Female were daily checked for the presence of a clutch. All clutches were incubated at 24 °C in an Exo Terra thermoelectric reptile egg incubator until hatching (Lindtke et al. 2010; Rodríguez-Díaz et al. 2010). After oviposition/parturition, females were released at point of capture.

Nine female reproductive traits were measured: clutch size (CS), clutch mass (CM), average egg mass (EM = CM/CS), relative clutch mass measured as clutch mass divided by female weight after oviposition or parturition (RCM), relative offspring mass measured as total sum of each offspring mass divided by female weight after oviposition or parturition (ROM), average offspring size (OS), average offspring mass (OM), average offspring body condition (OM/OS), and total offspring biomass. Female weight after oviposition/parturition, EM, RCM and ROM were only available for sampling year 2016, so sample sizes were smaller for these traits (total *N* = 165). RCM includes the mass of the whole clutch, including eggshell, amniotic fluids, yolk and the embryo. In a few cases offspring from viviparous females had already hatched before the clutch could be weighed; these clutches were excluded from RCM measures as they were lacking amniotic fluids and eggshells. ROM is the summed mass of the hatchlings, therefore, excluding eggshells, amniotic fluids and yolk remains. The number of infertile eggs (no embryo visible) and non-hatching offspring (embryos between stage 32 and 40 sensu Dufaure and Hubert 1961) was recorded and, together with the total number of offspring, was used to calculate hatching success, which is the proportion of the total hatched offspring relative to clutch size. Non-hatching offspring was divided into two classes, embryos that died early in development (stages 32–35) and late in development (stages 36–40). Here, we use the terms ‘clutch’ and ‘hatching’ for both oviparous and viviparous common lizards. An overview of mean values for all measured traits and differences between the two reproductive modes are summarized in Table 1.

**Table 1:** Sample sizes, mean and standard variation for all measured traits for oviparous and viviparous females

	Oviparous			Viviparous			Delta mean	% Difference	*F*	*η* ^2^	*P*	
	*N*	Mean	SD	N	Mean	SD						
Female SVL	235	56.96	4.78	193	61.96	5.66	5.01	8.8	93.0	0.17	< 0.0001	***
Female weight^a^	237	4.84	1.09	203	5.78	1.58	0.94	19.5	141.9	0.09	< 0.0001	***
Female weight^b^	79	3.67	0.77	76	4.22	0.72	0.55	14.9	37.5	0.13	< 0.0001	***
Weight loss	79	1.31	0.20	76	1.49	0.30	0.18	13.9	40.9	0.11	< 0.0001	***
Clutch size	232	6.73	2.04	204	5.81	1.75	0.92	15.9	41.2	0.05	< 0.0001	***
Offspring size	190	22.05	0.88	193	20.45	0.94	1.59	7.8	308.0	0.44	< 0.0001	***
Offspring weight	189	0.26	0.03	194	0.19	0.02	0.07	36.7	642.5	0.64	< 0.0001	***
Offspring body condition	189	0.12	0.01	192	0.09	0.01	0.02	26.8	658.3	0.64	< 0.0001	***
EM	79	0.25	0.05	76	0.35	0.08	0.10	38.7	87.9	0.34	< 0.0001	***
RCM	79	0.50	0.14	76	0.56	0.21	0.06	12.2	5.7	0.02	0.0183	NS
ROM	68	0.51	0.11	72	0.30	0.07	0.20	66.4	207.4	0.55	< 0.0001	***
Offspring biomass	189	1.33	0.54	194	0.92	0.36	0.41	44.9	101.5	0.13	< 0.0001	***
Infertility	230	0.17	0.34	206	0.06	0.20	0.11	186.4	15.8	0.03	< 0.0001	***
Early mortality^c^	230	0.06	0.16	206	0.01	0.06	0.04	321.1	15.3	0.04	0.0001	**
Late mortality^d^	230	0.03	0.10	206	0.09	0.22	0.05	61.5	16.3	0.03	< 0.0001	***
Hatching success	230	0.72	0.38	206	0.84	0.30	0.12	16.1	12.9	0.03	0.0004	**
Offspring hatched	232	4.23	2.77	206	4.58	2.22	0.34	8.1	2.1	0.02	0.1514	NS

Relative measures (EM, RCM, ROM, infertility, early mortality, late mortality, and hatching success) are shown as proportions. The absolute (delta mean) and proportional difference (% difference) between reproductive modes in each trait is specified. Finally, ANCOVA statistics in each trait between the reproductive modes are shown including significance after Bonferroni correction

*EM* egg mass, *RCM* relative clutch mass, *ROM* relative offspring mass, *NS* not significant

* *P* < 0.05; ** *P* < 0.01; *** *P* < 0.001

^a^Weight measured at time of captivity

^b^Weight measured after oviposition/parturition

^c^Embryos at developmental stage 32–35

^d^Embryos at developmental stage 36–40

### Statistical analyses

All statistics analyses were carried out in R vers. 3.2.3 (R Core team 2015). To test for difference in female body size (SVL) and weight between reproductive modes, we applied ANCOVAs with sampling year and altitude at point of collection as covariates. For differences in reproductive traits, female body size, number of days in captivity until parturition/gestation, sampling year, and altitude were added as covariates in an ANCOVA. Data were normalized prior to ANCOVAs and checked for heteroscedasticity using Levene’s test. Interactions between SVL × reproductive mode, sampling year × reproductive mode and altitude × reproductive mode were included in the model to test and correct for environmental variation and reproductive mode. Normal distribution of model residuals was checked using a Shapiro–Wilk test. We corrected for multiple testing by applying a Bonferroni correction. Results are summarized in Table S1. Models with a significant interaction between SVL and reproductive mode and a life-history trait were subsequently tested for reproductive mode-specific effects of body length (Table S2).

In addition to ANCOVAs, we performed a multivariate approach using a principal component analysis (PCA) in R. Principal component (PC) loadings were compared to assess the importance of variables relative to each other for each PC. Linear regressions were performed to check if PCs differed between reproductive modes.

## Results

### Female body size and body weight

**Fig. 2:**
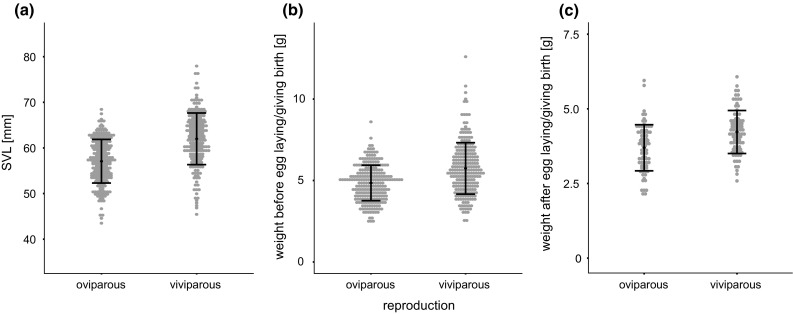
Body size (snout-vent length [SVL]) and weight of oviparous and viviparous female common lizards (*Zootoca vivipara*) from the contact zone at Straniger Alm in Austria. Mean and standard error are shown for each panel. Viviparous females are **a** larger and heavier **b** before and **c** after giving birth/egg-laying than oviparous females. The raw data are shown, uncorrected for effects such a body size or duration of captivity

Viviparous females were significantly larger than oviparous females, as measured by body length (snout-vent length [SVL]) (*N* = 428, *F* = 98.2, *η*^2^ = 0.19, *P* < 0.0001; Fig. 2a). On average, viviparous females were 4.9 mm larger (Table 1).

Viviparous females were heavier than oviparous females both before (*N* = 440, *df* = 1, *F* = 152.9, *η*^2^ = 0.10, *P* < 0.0001; Fig. 2b) and after parturition/oviposition (*N* = 155, *F* = 43.0, *η*^2^ = 0.13, *P* < 0.0001; Fig. 2c). On average, viviparous females were 0.55 g heavier than oviparous females after giving birth/laying eggs (Table 1).

### Offspring number, size and body condition at birth

**Fig. 3:**
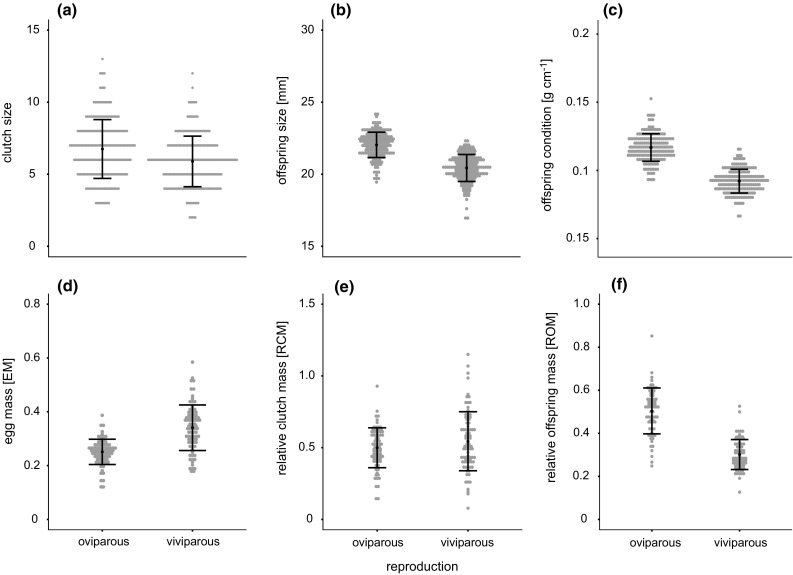
Reproductive trait variation between oviparous and viviparous common lizard females. The raw data are shown, uncorrected for effects such a body size or duration of captivity. Mean and standard error are indicated for each plot as oviparous females have **a** larger clutch sizes and larger **b** offspring size and **c** weight. The **d** egg mass (EM) is larger for viviparous females. **e** Relative clutch mass (RCM) is larger for viviparous females, but does not differ significantly after Bonferroni correction. Finally, **f** relative offspring mass (ROM) is larger for oviparous females

Clutches laid by viviparous females had on average almost one offspring fewer (*∆* = 0.92) than clutches laid by oviparous females (*N* = 436, *F* = 40.9, *η*^2^ = 0.05, *P* < 0.0001; Table 1; Fig. 3a; Table S1). In both reproductive modes, clutch size was highly correlated with SVL (oviparous: *N* = 228, *t* = 13.24, *R*^2^ = 0.434, *P* < 0.0001; viviparous: *N* = 188, *t* = 12.13, *R*^2^ = 0.44, *P* < 0.001).

Offspring from viviparous females were on average smaller in body length (*N* = 383, *F* = 324.0, *η*^2^ = 0.44, *P* < 0.0001; Fig. 3b) and weighed less (*N* = 383, *F* = 678.9, *η*^2^ = 0.64, *P* < 0.0001; Fig. 3c) than the offspring from oviparous females (Table 1). Body condition was higher in oviparous offspring compared to viviparous offspring (*N* = 381, *F* = 49.9, *η*^2^ = 0.12, *P* < 0.0001; Table 1; Table S1).

Offspring weight was positively correlated with the mother’s body size in viviparous females (size: *N* = 177, *t* = 3.38, *R*^2^ = 0.06, *P* < 0.001; Table S2) whereas this correlation was not significant in oviparous females (size: *N* = 190, *t* = 0.14, *R*^2^ < 0.01, *P* = 0.11; Table S2).

### Reproductive investment

On average, parturition in viviparous females occurred 42 days later than oviposition in oviparous females. The average egg mass (EM) was significantly larger in viviparous females (*N* = 155, *F* = 84.7, *η*^2^ = 0.33, *P* < 0.0001; Fig. 3d), indicating that clutches of viviparous females weighed more at the time of parturition than oviparous clutches did at the time of oviposition. EM is also positively correlated with female body size in viviparous females (*N* = 76, *t* = 3.64, *R*^2^ = 0.15, *P* < 0.001), suggesting that larger females invest in clutch mass and clutch size, i.e. number of offspring per clutch (Table S2).

However, at the time of oviposition/parturition, the relative clutch mass (RCM) in viviparous lizards was only slightly larger than that for oviparous lizards and did not differ significantly after Bonferroni correction between the two reproductive modes (*N* = 155, *F* = 4.48, *η*^2^ = 0.02, *P* = 0.04; Fig. 3e). This might be due to the reduced clutch size produced by viviparous females and their greater weight.

Finally, the summed offspring mass relative to the female weight (ROM) was significantly smaller for viviparous females (*N* = 140, *F* = 222.9, *η*^2^ = 0.57, *P* < 0.0001; Fig. 3f), indicating that oviparous females have a higher net output per clutch, also suggested by a larger total offspring biomass (*N* = 383, *F* = 112.9, *η*^2^ = 0.18, *P* < 0.0001; Table 1).

### Hatching success

Hatching success was higher for viviparous offspring compared to oviparous (*N* = 436, *F* = 9.9, *η*^2^ = 0.02, *P* = 0.0017) (Table 1). This was due to a lower percentage of infertile eggs (6.0% vs. 17.1%) and lower percentage of embryos that died at an early stage of development (1.2% vs. 5.8%) in viviparous clutches (Table 1). The number of hatched offspring did not differ significantly between oviparous and viviparous females (*N* = 438, *F* = 2.07, *P* = 0.237; Table 1).

### Principal component analysis (PCA)

**Fig. 4:**
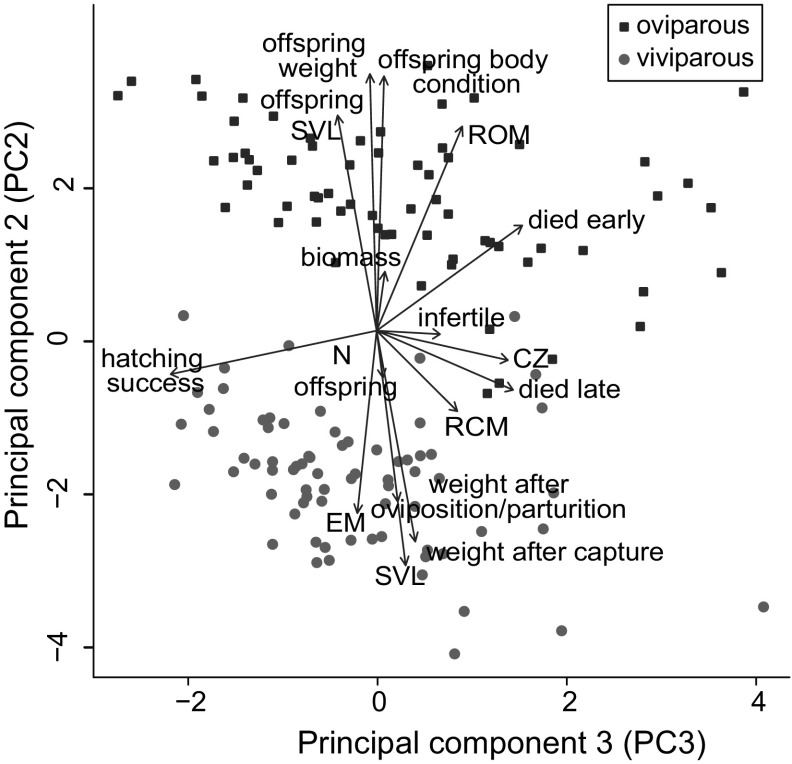
Principal component analysis (PCA) of female body size and reproductive traits. The plot shows principal components (PCs) 2 and 3. Both components significantly differ between oviparous (red squares) and viviparous (blue dots) females. Only individuals with complete data on body size and reproductive traits are included (*N* = 138)

The PCA summarized common associations between reproductive variables and the two reproductive modes. The first PC explained 29.9% of the variance, and mainly described reproductive variables (CS, offspring size, RCM, ROM, offspring biomass, number of offspring hatched, and hatching success) increasing with body size (SVL, weight; all loadings > 0.2; Table S3). PC1 differed significantly between reproductive modes (*N* = 138, *F* = 4.4, *η*^2^ = 0.03, *P* = 0.04), and viviparous females tended to have a higher score (mean oviparous = 0.40, mean viviparous = − 0.37) on that PC. Reproductive modes were significantly different on PC2, with almost no overlap between oviparous and viviparous individuals (*N* = 138, *F* = 551.9, *η*^2^ = 0.80, *P* < 0.0001; Fig. 4). PC2 explained 26.2% of the variance, and was associated with low SVL and weight, large offspring output (including large ROM, offspring SVL, weight, body condition and biomass), and low egg mass (Table S3). These are also the clearest differences (viviparity positively correlated with PC2) associated with the two reproductive modes in the ANCOVAs. PC3, explaining 11.7% of variance, also differed significantly between reproductive modes (*N* = 138, *F* = 5.85, *η*^2^ = 0.04, *P* = 0.017; Fig. 4). This PC was associated with large clutch size, RCM, ROM and a strong association with lower hatching success (with a low number of overall hatching offspring and a large proportion of non-hatching embryos) (Table S3).

## Discussion

Here, we show that reproductive investment strategies differ substantially between syntopically occurring, reproductively bimodal oviparous and viviparous common lizards. Of our five predictions, we found empirical support for four: viviparous females exhibit larger body size, smaller clutch sizes, a larger reproductive investment, and a higher hatching success rate than oviparous females (our predictions i, ii, iv, and v). However, contrary to our prediction iii, offspring size and weight from viviparous females was lower compared to offspring from oviparous females (Table 1). This may suggest an effect of space constraint during pregnancy. Female body size had a major impact on reproductive output, particularly in viviparous females. More reproductive traits were significantly associated with body length in viviparous females compared to oviparous females. The selective benefit of larger size may, therefore, facilitate increasing body size in the evolution of viviparous lineages. Reproductive output is lower for viviparous than for oviparous common lizards. While the production of larger offspring could offset the smaller clutch size in viviparous compared to oviparous females, this was not the case and suggests that reproductive output in viviparous common lizards is constrained by body size. We propose an adaptive scenario for life-history trait evolution following the transition from oviparity to viviparity across vertebrates.

### Body size evolution

We show that viviparous females have evolved larger body sizes compared to oviparous females. On average, viviparous females are almost 5 mm larger than oviparous females (Table 1). This agrees with the observation that viviparous are generally larger than oviparous species (Tinkle et al. 1970; Dunham and Miles 1985; Dunham et al. 1988; Cei et al. 2003), though this has received only weak support within a phylogenetic context (Meiri 2008). In both other reproductively bimodal lizard species, *Lerista bougainvillii* and *Saiphos equalis*, the viviparous form also exhibits larger body size than the oviparous (Qualls and Shine 1995, 1998; Smith and Shine 1997). Currently, we do not know whether these differences in size of common lizards stem from differences in growth rate, age at maturation, age structure and/or longevity. The impact of environmental factors on body size evolution has been reported for some reptile species but remains unresolved (Adolph and Porter 1993; Shine 2005; Horváthová et al. 2013; Pincheira-Donoso and Meiri 2013; Roitberg et al. 2013). The distribution of oviparous and viviparous lizards within the sampling site is not associated with any habitat-specific variable (Recknagel et al., unpublished). The only environmental variable possibly affecting reproductive traits is altitude (as a predictor for temperature), and was controlled for in all statistical tests (see Table S1). Altitude may play a role in adult habitat origin and selection but here all females laid their clutches at the same altitude. While phenotypic plasticity in size is unlikely to play an important role here, as the environment is similar, other selective pressures or genetic drift might have contributed to the evolution of the observed differences in body size between reproductive modes. Local adaptation—for example as a result of competition between the two reproductive modes—could be another mechanism driving divergence in body size, but seems unlikely given the substantial evolutionary divergence between them (Recknagel et al. 2018).

A significant interaction between body size and reproductive mode with a few life-history traits indicated that the effect of body size differs between reproductive modes (Table S2). In three out of five cases, the relationship was stronger in viviparous females compared to oviparous females (weight, offspring weight, and egg mass) (Table S2; Fig. S1). In contrast, clutch size and total biomass produced were more strongly correlated with SVL in oviparous compared to viviparous females. This suggests that reproductive output in oviparous females is mainly constrained by the number of eggs they can fit in their body, but not the increase in egg and offspring size during development, as this part of development occurs outside the mother. A strong association between body size and reproductive investment in viviparous relative to oviparous common lizards agrees with previous research (Horváthová et al. 2013) in this species. Here, as these common lizards are closely related geographic lineages, we show support for the body size hypothesis largely independent of phylogenetic bias.

### Difference in clutch size and reproductive investment

Both reproductive modes show a strong association between body size and clutch size, indicating that larger females generally produce larger clutch sizes. This is a well-established relationship in squamate reptiles (Dunham and Miles 1985; King 2000). Viviparous common lizards have significantly smaller clutch sizes, on average almost one individual less per clutch compared to oviparous clutches (Table 1). This agrees with previous studies in *Zootoca vivipara* (Lindtke et al. 2010; Roitberg et al. 2013), except for one study finding the opposite pattern (Horváthová et al. 2013). Offspring mass, weight and body condition were larger for the offspring of oviparous females, indicating a larger female investment, presumably through provision of more yolk. Clutches laid by oviparous females are much lower in weight at the time of oviposition compared to viviparous clutches at the time of parturition, as indicated by the clutch mass to size ratio (= relative egg mass; Table 1). The reason for this is that during egg development, the size of the egg increases substantially, mainly due to water uptake (Mathies and Andrews 1995; Qualls and Andrews 1999; Sun et al. 2012). While for oviparous clutches, this increase in weight occurs outside of the mother’s reproductive tract after oviposition, viviparous females must cope with their clutches’ increase in size and weight internally.

Pregnancy poses a reproductive burden to lizards, as they are less mobile and, therefore, more vulnerable to predation during this time (Shine 1980; Bauwens and Thoen 1981; Van Damme et al. 1989; Itonaga et al. 2012). The time of fertilization could not be measured in this study, but both reproductive modes become active around the same time in spring as soon as snow melts. Viviparous females are, therefore, probably affected by pregnancy about a month longer than oviparous females. The increase in mass at later stages of embryonic development poses an additional reproductive burden to viviparous common lizards, and we propose is compensated by smaller clutch size. This is also consistent with an increased oxygen consumption by viviparous females in the last third part of the pregnancy (Foucart et al. 2014). In poeciliid fishes, viviparity has also been associated with smaller clutch sizes relative to oviparous (Thibault and Schultz 1978; Mank and Avise 2006). Across reptiles, the pattern is somewhat unclear, with large-scale studies suggesting generally larger clutch sizes for viviparous reptiles (Tinkle et al. 1970; Iverson 1987). An explanation for this perhaps non-intuitive increase in viviparous clutch size might be that several oviparous species have multiple clutches per year, while viviparous species only have a single clutch per year, and single-brooded species have larger clutch sizes than multi-brooded species (Tinkle et al. 1970). For example, in common lizards, oviparous lizards at lower altitudes often lay two or more clutches (Heulin et al. 1991, 1994; Roig et al. 2000) whereas viviparous usually only lay one clutch per year (Bestion et al. 2015), mainly depending on climatic conditions. There might be less selective pressure for increasing clutch size in oviparous species, while single-brooded viviparous clutch size should be maximized and might be under stronger selective pressure (Cox et al. 2003; Roitberg et al. 2013). However, at high altitudes such as studied here, oviparous common lizard populations also usually produce a single clutch per year (Rodríguez-Díaz and Braña 2012).

We found another measure of reproductive investment, the relative clutch mass (i.e. clutch mass relative to mother’s weight; RCM), was slightly larger in viviparous females. This is in agreement with a study on the reproductively bimodal lizard *L. bougainvillii,* in which RCM was larger for viviparous compared to oviparous females (Qualls and Shine 1998). In contrast, in reproductively bimodal *S. equalis* the oviparous form exhibits a larger RCM; however, the differences in clutch size and mass do not differ to the same degree as observed in the other two reproductively bimodal species, presumably as both modes overlap in their egg retention time and embryos are close to full development (stages 38–39) (Smith and Shine 1997). Decreasing clutch size and increasing body size (= larger body cavity allowing for more space with developing offspring) are two ways viviparous common lizards can accommodate the additional reproductive burden. Comparisons across oviparous and viviparous common lizard populations also showed higher reproductive investment for viviparous females (Horváthová et al. 2013; Roitberg et al. 2013). In summary, reproductive investment differed between reproductive modes, and the direction of which mode invested more also depended on the type of investment (e.g., yolk provision, water provision, length of pregnancy). We suggest that the larger body size and lower clutch size in viviparous common lizards follow from the increased reproductive burden.

### Offspring size, weight, survival and total reproductive output

Contrary to our expectation, offspring size and weight was dramatically reduced in viviparous females, with offspring from viviparous mothers being more than 35% lighter than oviparous offspring. This strong association was also clear from the PCA, in which PC2, which mainly differentiated the two reproductive modes, had the highest loadings for offspring size, weight and body condition (Fig. 4). Previous research across the distribution of *Zootoca vivipara* also indicated that offspring size was smaller in viviparous populations (Lindtke et al. 2010; Roitberg et al. 2013). This is contrary to our prediction because a trade-off between clutch size and offspring size should result in either more numerous, smaller offspring or fewer, but larger offspring (Stearns 1976; Reznick 1982; Sinervo and Licht 1991; Olsson and Shine 1997); here clutch number and offspring size were both reduced in viviparous reproduction. For example, viviparous fishes usually produce fewer, but larger offspring that have increased survivorship compared to smaller offspring (Reznick 1982; Heath and Blouw 1998; Goodwin et al. 2002; Shikano and Taniguchi 2005). A relationship between offspring size and survivorship has also been demonstrated for reptile species (Sinervo 1990; Webb et al. 2006; Pike et al. 2008). However, a clear pattern of reproductive mode and offspring size has not been demonstrated in reptiles (Vitt and Blackburn 1983; Seigel and Fitch 1984; Lindtke et al. 2010; Sun et al. 2012). One study showed that viviparous offspring tend to have higher survivorship compared to oviparous offspring, though this pattern was not robust when accounting for phylogeny (Pike et al. 2008). Our study provides new data in this regard.

In accordance with other studies, we found that viviparous females had a lower reproductive output compared to oviparous females (Seigel and Fitch 1984; Meiri et al. 2012), but a higher hatching success. Indeed, we found that the greater hatching success compensated for the lower clutch size, so that between reproductive modes there was no significant difference in the number of offspring after hatching. Hatching success was generally high for both reproductive modes and comparable to previous estimates (Massot et al. 1992; Vercken et al. 2007). Infertility and mortality at early embryonic development was higher for oviparous clutches (Table 1; Table S1). Death at early embryonic development for eggs might be explained by a higher loss to fungal or microbial infections compared to embryos developing inside the mother and protected by its immune system (Blackburn and Evans 1986). Conditions were held constant for oviparous clutches, while viviparous embryos presumably experienced more temperature fluctuations in the course of the experiment. These are expected to differ between sampling years with natural weather variation; however, we did not observe a significant interaction between sampling year and reproductive mode for hatching success (Table S1).

While offspring mass and reproductive output was much greater for oviparous females, this difference may be augmented in our study because eggs were incubated at stable and slightly higher temperatures than ambient and it is known that environmental temperatures can influence size and viability of offspring (Van Damme et al. 1992; Shine and Harlow 1996; Shine 2002, 2005; Li et al. 2017). Particularly, the lower temperatures at higher altitudes have a negative effect on clutch development in oviparous females but can be mitigated by viviparous females (Shine 2002; Webb et al. 2006). At the sampling site (> 1300 m), egg incubation time under natural conditions is not known but would be substantially longer than in our study (Rodríguez-Díaz and Braña 2011, 2012). Further, hatching success for oviparous clutches was likely higher under our incubation conditions than under strictly natural settings. We suggest this is compatible with the expectation that at optimal developmental temperatures oviparous reproduction has an advantage, whereas under cold environmental conditions this advantage reverses (in agreement with the cold climate hypothesis (Tinkle and Gibbons 1977)).

We found a significant effect of female body size on offspring size and weight in viviparous females, but not in oviparous females (Table S2). This again suggests that female cavities are size limited, and only larger females can provide enough space for the development of larger offspring. This is compatible with a strong selective pressure for increased body size (Shine 2005). In oviparous females, much of the offspring mass is acquired after oviposition and is, therefore, not directly constrained by female size though perhaps by other environmental and physiological effects.

Differences in offspring traits can result from different temperatures exposed to during incubation, both if experienced within the mother’s reproductive tract or outside. For example, it has been shown that incubation temperature affects offspring head length and survival in oviparous *Zootoca vivipara* (Heulin et al. 1994; Rodríguez-Díaz et al. 2010). Also, temperatures experienced by egg clutches in the environment usually show large amounts of variation, with changes of more than 10 °C variation being common (Rodríguez-Díaz et al. 2010). While we tried to minimize effects resulting from incubation temperature consistency in our experiment and keep them as close as possible to temperatures experienced in the natural environment, we cannot exclude that some of the observed differences in offspring traits include an effect of incubation temperature. We accounted for the duration of days kept in captivity for each lizard using it as a covariate in our statistical analyses. Environmental conditions experienced in captivity were generally comparable to conditions at the sampling site, as lizards had the option to thermoregulate and bask depending on external weather conditions. In general, our observed differences between viviparous and oviparous offspring traits match those observed by other studies (Lindtke et al. 2010; Roitberg et al. 2013). However, we note that rearing conditions do not (and cannot) perfectly match conditions experienced at the nearby sampling site.

In summary, our results suggest that viviparous females are substantially constrained by body size. This has a negative effect on offspring size and weight. We propose that part of this effect can be compensated by a higher hatching success for viviparous clutches compared to oviparous clutches and costs of oviparity given the lower temperatures of high altitude sites.

### An evolutionary scenario for the association between life-history traits and viviparity

Alternative trade-off strategies between divergent life-histories are common across the animal kingdom (Stearns 1976; Reznick et al. 1990; Shine 2005; Pires et al. 2011). In reproductively bimodal common lizards, viviparous females must deal with a prolonged pregnancy and the increase of body mass during embryonic development (Qualls and Andrews 1999). In addition, the increase in weight and duration of pregnancy poses another problem to viviparous females: Their sprint speed and endurance is greatly reduced which increases predation risk (Shine 1980). It has been shown that gravid common lizard females behaviourally shift to a cryptic strategy rather than escape tactics when a predator approaches (Bauwens and Thoen 1981; Van Damme et al. 1989). The costs of pregnancy and space constraints for development are a reproductive burden affecting the total physical available space for reproducing viviparous females. On a long-term evolutionary scale, females could counteract this constraint via several adaptations: (i) an increase in body size, allowing for a larger body cavity; (ii) a reduction in clutch size; (iii) a reduction in offspring size and mass; and (iv) an increase in hatching success. Alternatively, viviparous females could be generally less productive than oviparous females, for example as a result of environmentally less favourable conditions experienced (Meiri et al. 2012).

On the oviparity–viviparity continuum, the common lizard is the evolutionarily youngest transition to viviparity known to date (Surget-Groba et al. 2006). While the benefits of being viviparous in cold environments are evident, the suite of life-history traits associated with viviparous reproduction in squamates might vary across different evolutionary stages. Organisms that evolved viviparity deeper in their evolutionary history have had more time to optimize their reproductive output than species with more recent transitions to viviparity. For example, the reproductively bimodal lizard, *Lerista bougainvillii*, evolved viviparity earlier than common lizards (Qualls and Shine 1998; 14.7 mya (5.8–23.6 mya 95% HPD) Recknagel et al. unpublished) and viviparous *L. bougainvillii* have a body size on average 10.0% greater relative to oviparous *L. bougainvillii*, which is compatible with adaptation for larger body cavities for a higher clutch size and/or mass (Qualls and Shine 1998). At present, viviparous common lizards have a body size that is 8.6% larger relative to syntopic oviparous females (Table 1). Unlike viviparous common lizards and most viviparous species (Seigel and Fitch 1984; Qualls and Shine 1995), viviparous *L. bougainvillii* do not differ in clutch size compared to oviparous. Therefore, we propose that in the common lizard constraint for space may be the main limiting factor decreasing reproductive output.

Another possible life-history adjustment to accommodate a decreased productivity per season would be an increase in lifetime number of reproductive events. A link between longevity and reproductive mode has been suggested generally (e.g., Tinkle et al. 1970; Stearns 1976; Gunderson 1997). In support of that, a comprehensive analysis found that larger squamates with few, large offspring tend to live longer than smaller squamates that reproduce more frequently, have larger clutch sizes, and smaller offspring; however, while reproductive mode correlates with some of these life-history traits, it did not have a significant effect on lifespan (Scharf et al. 2015). It is not known whether common lizard reproductive modes differ in their lifespan, and future research should address this question.

We conclude that the link between reproductive life-history traits and reproductive mode depends on several aspects. The degree of correlation may depend on the time since the transition in reproductive mode, from oviparity to viviparity, occurred. We do not imply here that traits correlated with viviparity evolved after the origin of viviparity; some of these traits might have evolved prior to the arising of viviparity. Also, even if reproductive traits had enough time to co-evolve to a more advantageous combination, different environmental conditions might favour different sets of correlated traits at different sites and across time, not always leading to the same direction in each reproductive trait (e.g., Medina and Ibargüengoytía 2010; Meiri et al. 2012; Sun et al. 2012; Bassar et al. 2014). Reproductive traits represent a life-history trade-off that is context dependent. Finally, life-history traits can also be directly influenced by differences in incubation conditions during embryonic development, suggesting that the evolution of viviparity may have been promoted by selection on offspring phenotypes (Li et al. 2017). Testing groups at different stages along the oviparity–viviparity continuum and across different environments, with phylogenetic correction, might give a clearer picture on the ecology of reproductive traits and reproductive mode.

## Electronic supplementary material

Below is the link to the electronic supplementary material.

Supplementary material 1 (PDF 1291 kb)
