# Supplementary material for: Differential reproductive investment in co-occurring oviparous and viviparous common lizards (Zootoca vivipara) and implications for life-history trade-offs with viviparity
Source: Oecologia. 2019 May 6;190(1):85–98. doi: 10.1007/s00442-019-04398-w (PMC6535419; doi:10.1007/s00442-019-04398-w)

Differential reproductive investment in co-occurring oviparous and viviparous common lizards (*Zootoca vivipara*) and implications for life-history trade-offs with viviparity

Hans Recknagel and Kathryn R. Elmer

Supplementary Information

Table S1. Summary statistics of all statistical models including all effects tested. Response variables are listed first from a) to p). Note that year was only included as an effect when data was collected from more than one sampling year. Significant p-values (P < 0.05) are indicated in italics, and significance after Bonferroni correction is specified in the last column.

effect	Mean sq	F	η^2	P-value	sign.
**a) body size**					
parity mode	1.410	98.20	0.19	*<0.0001*	***
year	0.061	4.28	0.01	*0.0393*	NS
captivity duration	0.067	4.69	0.01	*0.0309*	NS
altitude	0.131	9.11	0.02	*0.0027*	*
year x parity mode	0.008	0.54	0.00	0.4629	NS
altitude x parity mode	0.006	0.42	0.00	0.5194	NS
**b) body weight before egg laying/giving birth**					
parity mode	0.701	152.90	0.10	*<0.0001*	***
SVL	3.773	823.50	0.54	*<0.0001*	***
SVL x parity mode	0.123	26.84	0.02	*<0.0001*	***
year	0.005	1.03	0.00	0.3121	NS
captivity duration	0.461	100.66	0.07	*<0.0001*	***
altitude	0.016	3.42	0.00	0.0651	NS
year x parity mode	0.000	0.07	0.00	0.7972	NS
altitude x parity mode	0.019	4.16	0.00	*0.0420*	NS
**c) body weight after egg laying/giving birth**					
parity mode	0.780	42.95	0.13	*<0.0001*	***
SVL	2.024	111.50	0.34	*<0.0001*	***
SVL x parity mode	0.000	0.01	0.00	0.9450	NS

effect	Mean sq	F	η^2	P-value	sign.
**i) RCM**					
parity mode	0.104	4.48	0.02	*0.0359*	NS
SVL	0.489	21.11	0.11	*<0.0001*	***
SVL x parity mode	0.042	1.82	0.01	0.1800	NS
captivity duration	0.205	8.83	0.05	*0.0035*	NS
altitude	0.002	0.06	0.00	0.8002	NS
altitude x parity mode	0.013	0.54	0.00	0.4631	NS
**j) ROM**					
parity mode	2.689	222.94	0.57	*<0.0001*	***
SVL	0.345	28.61	0.07	*<0.0001*	***
SVL x parity mode	0.018	1.47	0.00	0.2273	NS
captivity duration	0.075	6.22	0.02	*0.0139*	NS
altitude	0.008	0.68	0.00	0.4129	NS
altitude x parity mode	0.003	0.21	0.00	0.6466	NS
**k) offspring biomass**					
parity mode	1.977	112.93	0.18	*<0.0001*	***
SVL	2.643	150.98	0.24	*<0.0001*	***
SVL x parity mode	0.218	12.45	0.02	*0.0005*	**
year	0.000	0.02	0.00	0.8868	NS
captivity duration	0.141	8.04	0.01	*0.0048*	NS

captivity duration	0.377	20.74	0.06	*<0.0001*	***
altitude	0.004	0.23	0.00	0.6350	NS
altitude x parity mode	0.005	0.29	0.00	0.5940	NS
**d) clutch size**					
parity mode	0.703	40.91	0.05	*<0.0001*	***
SVL	5.428	316.04	0.40	*<0.0001*	***
SVL x parity mode	0.142	8.27	0.01	*0.0043*	NS
year	0.177	10.32	0.01	*0.0014*	*
captivity duration	0.008	0.49	0.00	0.4833	NS
altitude	0.081	4.73	0.01	*0.0302*	NS
year x parity mode	0.000	0.00	0.00	0.9613	NS
altitude x parity mode	0.032	1.86	0.00	0.1732	NS
**e) offspring size**					
parity mode	4.622	323.97	0.45	*<0.0001*	***
SVL	0.129	9.07	0.01	*0.0028*	*
SVL x parity mode	0.037	2.56	0.00	0.1104	NS
year	0.000	0.02	0.00	0.9000	NS
captivity duration	0.344	24.09	0.03	*<0.0001*	***
altitude	0.001	0.04	0.00	0.8489	NS
year x parity mode	0.009	0.63	0.00	0.4270	NS
altitude x parity mode	0.013	0.94	0.00	0.3329	NS
**f) offspring weight**					
parity mode	7.148	678.90	0.64	*<0.0001*	***
SVL	0.007	0.63	0.00	0.4271	NS
SVL x parity mode	0.056	5.30	0.01	*0.0219*	NS

altitude	0.055	3.16	0.01	0.0762	NS
year x parity mode	0.002	0.11	0.00	0.7454	NS
altitude x parity mode	0.019	1.06	0.00	0.3050	NS
**l) infertility**					
parity mode	2.710	14.21	0.03	*0.0002*	**
SVL	0.080	0.42	0.00	0.5178	NS
SVL x parity mode	0.355	1.86	0.00	0.1730	NS
year	0.217	1.14	0.00	0.2870	NS
captivity duration	0.713	3.74	0.01	0.0539	NS
altitude	1.854	9.72	0.02	*0.0020*	*
year x parity mode	0.894	4.69	0.01	*0.0310*	NS
altitude x parity mode	0.030	0.16	0.00	0.6932	NS
**m) early embryo mortality (stage 32-35)**					
parity mode	0.300	13.14	0.03	*0.0003*	**
SVL	0.034	1.50	0.00	0.2211	NS
SVL x parity mode	0.045	1.98	0.00	0.1603	NS
year	0.246	10.75	0.02	*0.0011*	*
captivity duration	0.128	5.60	0.01	*0.0184*	NS
altitude	0.017	0.73	0.00	0.3949	NS
year x parity mode	0.198	8.66	0.02	*0.0034*	NS
altitude x parity mode	0.000	0.00	0.00	0.9715	NS
**n) late embryo mortality (stage 36-40)**					
parity mode	0.498	15.09	0.03	*0.0001*	**
SVL	0.001	0.03	0.00	0.8538	NS
SVL x parity mode	0.006	0.17	0.00	0.6823	NS

year	0.000	0.00	0.00	0.9815	NS
captivity duration	0.154	14.65	0.01	*0.0002*	**
altitude	0.004	0.34	0.00	0.5582	NS
year x parity mode	0.078	7.37	0.01	*0.0070*	NS
altitude x parity mode	0.018	1.73	0.00	0.1889	NS
**g) offspring body condition**					
parity mode	2.413	49.91	0.12	*<0.0001*	***
SVL	0.589	12.19	0.03	*0.0005*	**
SVL x parity mode	0.080	1.65	0.00	0.1995	NS
year	0.002	0.03	0.00	0.8547	NS
captivity duration	0.048	0.99	0.00	0.3214	NS
altitude	0.013	0.27	0.00	0.6065	NS
year x parity mode	0.118	2.45	0.01	0.1185	NS
altitude x parity mode	0.003	0.06	0.00	0.8135	NS
**h) EM**					
parity mode	1.606	84.71	0.33	*<0.0001*	***
SVL	0.220	11.61	0.05	*0.0008*	*
SVL x parity mode	0.128	6.74	0.03	*0.0104*	NS
captivity duration	0.100	5.29	0.02	*0.0228*	NS
altitude	0.011	0.57	0.00	0.4505	NS
altitude x parity mode	0.000	0.02	0.00	0.8981	NS
year	0.495	15.01	0.03	*0.0001*	**
captivity duration	0.891	27.00	0.06	*<0.0001*	***
altitude	0.072	2.17	0.01	0.1411	NS
year x parity mode	0.199	6.03	0.01	*0.0145*	NS
altitude x parity mode	0.140	4.23	0.01	*0.0404*	NS
**o) hatching success**					
parity mode	3.035	9.94	0.02	*0.0017*	*
SVL	0.089	0.29	0.00	0.5892	NS
SVL x parity mode	0.259	0.85	0.00	0.3574	NS
year	1.509	4.94	0.01	*0.0268*	*
captivity duration	11.509	37.69	0.08	*<0.0001*	***
altitude	0.346	1.13	0.00	0.2879	NS
year x parity mode	0.050	0.16	0.00	0.6872	NS
altitude x parity mode	0.072	0.23	0.00	0.6286	NS
**p) offspring hatched**					
parity mode	0.050	1.41	0.00	0.2366	NS
SVL	2.599	72.67	0.14	*<0.0001*	***
SVL x parity mode	0.029	0.80	0.00	0.3710	NS
year	0.052	1.45	0.00	0.2298	NS
captivity duration	0.668	18.67	0.04	*<0.0001*	***
altitude	0.198	5.52	0.01	*0.0193*	NS
year x parity mode	0.000	0.00	0.00	0.9603	NS
altitude x parity mode	0.000	0.01	0.00	0.9338	NS

* P < 0.003; ** P < 0.0006; *** P < 0.00006; NS = not significant. Abbreviations are: EM = egg mass; RCM = relative clutch mass; ROM = relative offspring mass.

Table S2. Effect of SVL (snout vent length) on female weight and reproductive traits. Only models that included a significant interaction between SVL and parity mode (see Table S1) are included in this Table. The estimate indicates the slope and direction of the trend. Significant correlations are indicated with an asterisk (see below).

**trait**	**oviparous SVL**				**viviparous SVL**			
	N	estimate	P		N	estimate	P	
female weight	234	0.155	<0.001	***	187	0.234	<0.001	***
clutch size	228	0.286	<0.001	***	188	0.200	<0.001	***
offspring weight	186	-0.001	0.108	NS	178	0.001	0.015	*
egg mass	76	0.001	0.339	NS	75	0.008	<0.001	***
offspring biomass	186	0.058	<0.001	***	178	0.036	<0.001	***

* P < 0.05; ** P < 0.01; *** P < 0.001; NS = not significant

Table S3. Loadings for the first six principal components. These explained a total variance of 91%. PC1 was strongly associated with allometric effects and direct correlations between body size and reproductive traits, but also significantly associated with reproductive mode. PC2 strongly differentiated between traits that differed between reproductive mode, such as female body size, offspring traits, and EM. PC3 also differed significantly between reproductive modes, and had the highest loadings for hatching success, embryo mortalities, clutch size, RCM and ROM.

trait	PC1	PC2	PC3	PC4	PC5	PC6
SVL	0.22	-0.37	0.08	-0.18	0.18	-0.01
weight (capture)	0.25	-0.33	0.11	-0.22	0.15	0.00
weight (oviposition/parturition)	0.19	-0.27	0.06	-0.56	0.00	0.03
clutch size	0.35	-0.05	0.38	0.01	-0.27	0.07
offspring size	0.21	0.34	-0.11	-0.18	0.27	-0.03
offspring weight	0.18	0.40	-0.02	-0.15	0.27	-0.11
offspring body condition	0.16	0.40	0.02	-0.14	0.25	-0.13
EM	0.05	-0.28	-0.06	0.20	0.64	-0.10
RCM	0.23	-0.13	0.23	0.54	0.30	-0.06
ROM	0.27	0.32	0.24	0.24	-0.09	-0.04
offspring biomass	0.43	0.09	0.02	0.01	-0.15	-0.04
infertility	-0.10	-0.01	0.18	-0.20	-0.08	-0.84
early embryo mortality	-0.15	0.16	0.42	-0.22	0.21	0.47
late embryo mortality	-0.19	-0.09	0.39	0.19	-0.08	-0.15
hatching success	0.25	-0.07	-0.59	0.12	-0.09	-0.01
offspring hatched	0.42	-0.07	0.02	0.07	-0.26	0.04

Figure S1. Body size (SVL) in relation to life-history traits. Relationships in different reproductive modes are illustrated in red squares (oviparous) and blue circles (viviparous). In all life-history traits shown, the interaction between reproductive mode and SVL differed significantly (before Bonferroni correction; Table S1).

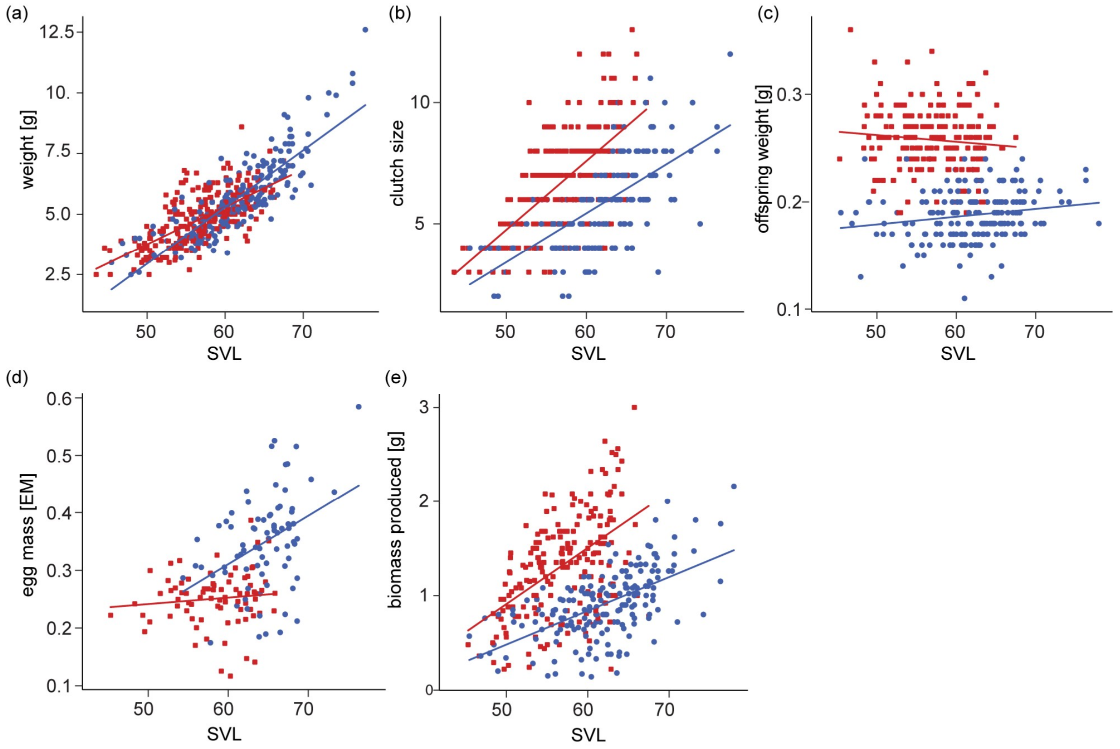

Supplement: Supplementary file 1 — Supplementary material 1 (PDF 1291 kb) [file 442_2019_4398_MOESM1_ESM.pdf]
